# How youth engage in online deliberation: an empirical study based on individual psychological motivations from China

**DOI:** 10.3389/fpsyg.2025.1546168

**Published:** 2025-08-01

**Authors:** Yuyang Lin, Yunpeng Tan, Xiao Wang, Zhenwei Liang

**Affiliations:** ^1^School of Journalism and Communication, Xiamen University, Xiamen, Fujian, China; ^2^General Office, Xiamen University, Xiamen, Fujian, China

**Keywords:** youth, public deliberation, involvement, inter-group emotional contagion, selective exposure

## Abstract

Clarifying the youth expression patterns on the internet and guiding contemporary youth to participate in public deliberation in an orderly manner within the online society will contribute to their growth and development and further promote the democratic development of society. While many studies have explored the impact of structural factors in the online environment on public deliberation, they overlook the psychological processes of the participants themselves. This paper, based on the background of social conflict events, focuses on the online public deliberation behavior of young people and explores how the involvement and inter-group emotional contagion influence the level of online public deliberation from the perspective of individual psychological motivations, as well as the mediating role of selective exposure. Through a questionnaire survey (*n* = 1,092), this study found that involvement has a positive impact on the level of online public deliberation, but similar to inter-group emotional contagion, it can lead to a higher degree of conversational dominance. Inter-group emotional contagion is not conducive to deliberation. Selective exposure serves as an important mediator between individual cognition and emotion and online public deliberation. The research findings examine the influence pathways of individual cognition on online public deliberation, providing insights for understanding the mechanisms of youth online expression and enhancing the degree of online public deliberation.

## 1 Introduction

Nowadays, China's Internet penetration rate has reached 76.4% (CNNIC, 2023), and with the rise of online society, a new form of interaction has been shaped by the technical characteristic of universal connectivity. Besides, Internet technology has created a virtual space, providing ideal conditions for democratic deliberation and serving as the infrastructure for public deliberation. As a result, online deliberation and online democracy have flourished with the deep integration of technology. However, on the one hand, Internet empowerment has made expressions freer and more convenient. On the other hand, it has also complicated public opinion ecology, resulting in social consensus being torn apart at times, leading to problems such as extreme flattening of public discussion, the rise of anti-intellectualism, which embodies features of binary opinion polarization, squeezing of rational discussion space, and tearing apart of consensus. Social change drives the theoretical deepening of online public deliberation.

At present, the number of 10-19-year-old netizens in China has reached 150,000,000 while 20-29-year-old is 156,000,000 (CNNIC, 2023), which indicates that youth stand as not only the main force in Internet use, but also the main participants in Internet political participation, expression, and deliberation. Therefore, through exploring and clarifying the intrinsic motivations of youth taking part in online public deliberation, we could effectively guide them to orderly participate in public deliberation in the deeply mediated digital age. It would benefit in cultivating young people's awareness of political participation and sense of ownership and building a peaceful cyberspace community. However, in recent years, though the relevant theoretical and empirical studies on online public deliberation have been growing continuously, many scholars have pointed out that this concept still needs to be developed, especially the individual motivations that support and promote online public deliberation (Friess and Eilders, 2015). Besides, most of these research focuses on the effect of network structure and its products on online public deliberation, namely the influence of social media and other digital platforms on the degree of public deliberation. In other words, it lacks insight into the psychological activities of the actors who participate in the deliberation and limiting the types of motivations that have been tested. At the same time, the difference of the degree of online public deliberation in different contexts is also lack of discussion, whose type, nature, intensity, and opinion climate would affect individual expression. The degree of online public deliberation which means the degree of online deliberative conversation behavior (Moy and Gastil, 2006).

Therefore, from the perspective of individual psychological motivation which includes cognitive, emotional, and volitional processes, this study will build a mechanism model of Chinese youth participation in online public deliberation when faced with social conflict events. This study try to expand the factors affecting the degree of online public deliberation from the psychological level of the actors and probe into its mechanistic pattern.

## 2 Materials and methods

### 2.1 Literature review and theory

#### 2.1.1 Motivation of online public deliberation

As an enduring academic concept and social ideal, public deliberation originated from Habermas' reflection on the historical development and communication behavior of the public sphere. With the deepening of research, the connotation and operational definition of public deliberation have constantly evolved, which includes: (1) reasonable expression, where participants support their expressions with reasonable and effective claims (Habermas, 1985), including the relevance of the discussion topic (Stromer-Galley, 2003); (2) communication of different opinions, where participants with different views interact with each other (Thompson, 2008); (3) understanding opposite views, where participants reflect on the claims of others and themselves, even opposing their own claims (Dahlberg, 2001); (4) clear expression of views, where participants express their views clearly (Moy and Gastil, 2006); (5) civil communication, where participants interact with each other in a polite and respectful manner (Papacharissi, 2004). What's more, the essence of the Internet is an open and integrated infrastructure for information dissemination and cultural exchange, which exhibits the characteristics of the public sphere and could approximately meet the level of public deliberation, providing ideal conditions for democratic deliberation.

Therefore, online public deliberation has become one of the most influential concepts in the discussion between Internet technology and democracy. With respect to its space, the earliest online public sphere usually appeared in the comment sections of online news (Ruiz et al., 2011), while the rise of social media brought the main venue for deliberation. Besides, scholars have discussed the participation motivations, structural mechanisms, communication quality, and individual changes and consensus generation in online public deliberation. Particularly they probe into the process and platform structure design of it, while the discussion of the “input” and “output” parts of online public deliberation still remains incomprehensive. Hence, scholars have called for future research to concentrate on examining the psychological motivations of participants at the individual level, as well as exploring the results of deliberation to fill the research gap (Friess and Eilders, 2015).

Furthermore, the willingness and motivation to participate in online public deliberation can be investigated from a dual perspective of structure and action. For one thing, from the network structure where public deliberation is located, network communication patterns (Janssen and Kies, 2005), anonymity (Leshed, 2009), censorship (Wise et al., 2006), discussion scale (Himelboim, 2008), and discussion heterogeneity (Zhang et al., 2013) and so on all have an impact on the willingness and the degree of discussion of online public deliberation. For another, centering on the intrinsic motivations of social actors, Gastil and Broghammer (2021) have based on “Maslow's hierarchy of needs,” believing that online public deliberation satisfies the motivations of participants to obtain material benefits, establish social relationships, and enhance self-esteem and social status. And then Wu et al. (2023) based on the theory of “Uses and Gratifications,” found that the use of social motivation, expression motivation, and information motivation has a positive effect on online public deliberation, while the negative use of entertainment has a negative effect. However, there is a lack of study on the participation motivations of online public deliberation, so that inspecting the relationship between diverse types of motivations and the degree of online public deliberation, and investigating the differences in different contexts and topics, has turned into a direction for the expansion of online public deliberation research. In other words, young netizens as social actors not only maintain structures but also change structures.

#### 2.1.2 Individual psychological motivation and network information behavior

Motivation refers to the inner force of behavior, initiating and guiding behavior and determining its intensity and persistence, whose essence have been investigated from various perspectives, forming different theories. Cognitive theory of motivation holds that people's decision-making about behavior is active, during which cognitive variables such as expectations, attention, and evaluation play an important role. In other words, cognition has a motivational function, that is, individuals process external information and form different beliefs in their minds, which act as mediators between stimuli and behavior, both causing and changing behavior. Moreover, individual cognitive level would affect their processing and understanding of information in the network as well as their communication and interaction. That's why many scholars have applied motivated cognition to the exploration of network information behavior.

Besides, emotion also serves as a source of motivation, a basic component of the motivation system. Since the cognitive revolution in psychology in the 1970s, the role of emotion in motivation has been valued, and then the affect theory has been highly regarded since the 1990s. Emotion is a positive force with motivation and perception that organizes, maintains, and guides behavior. In addition, human emotions not only spread in real-world communities, but also spread in online social media, driving information diffusion, individual connections, and network expression (Fan et al., 2016).

Will is closely related to cognition, emotion, which has the function of motivation and control to trigger behavior, playing a mediating effect on the interaction among human cognition, emotion, and behavior. In detail, Kuhl (1985) believes that as a goal-oriented activity. Volitional action, has a motivational role in the decision-making and goal-setting stages, constantly evaluating the sense of efficacy and value; while before fulfilling the action plan, the process of protecting the action intention to avoid being replaced by other competing tendencies is action control, also known as volitional control. Moreover, will could effectively control an individual's online information behavior. For example, when faced with emergencies, Chinese netizens' perceived behavior control and information dissemination behavior intention are positively correlated (Zhai et al., 2016).

In general, human behavior is always under the co-influence of cognitive process, emotional process, and volitional process, so that the independent role of cognitive, emotional or volitional processes in behavior. To sum up, this study will adopt a motivation-based perspective, building a behavioral mechanism model of youth participation in online public deliberation from the basic psychological activities of cognition, emotion, and volition when faced with emergent social conflict events, with the aim of exploring the influencing factors and mechanisms of online public deliberation.

### 2.2 Hypotheses and model

#### 2.2.1 Involvement degree and the degree of online public deliberation

Involvement could be understood as the subjective experience state of an individual's perception of the importance or relationship with an activity or something (Zaichkowsky, 1985), describing the degree of engagement of an individual in a certain cognitive task or activity. It can be regarded as a driving force to encourage them to participate in cognitive activities and invest more cognitive resources. In this study, involvement degree specifically refers to the audience's subjective experience of the importance and relationship with social conflict events, which leads to their different levels of attention to these events. It also represents the psychological process of young people's cognition of social conflict events, and the cognitive motivation for participating in online public deliberation.

Furthermore, previous studies have confirmed that the involvement degree closely relates to the information transmission and communication process of individuals in the network. The involvement degree could affect the way individuals process and participate in network information (Ziegele et al., 2018). Social cognition theory holds that individual factors influencing behavior come from outcome expectation and self-efficacy (Bandura, 1999). Outcome expectation includes the correlation between behavior and individual (involvement degree), so that involvement turns into the motivation of online public deliberation behavior. Then Bimber et al. (2012) investigated how people's attitudes, motivations, goals and social media use are related to collective action, arguing that the involvement degree embodies the degree of individual participation in organizational agenda setting and decision-making. That is, individuals with high involvement are more inclined to take the initiative to advocate or public deliberation.

As a result, involvement in events acts as the motivation to participate in online public deliberation. However, there is a lack of discussion between the involvement degree and deliberation in existing research, while the involvement degree may have an impact on the degree of online public deliberation. Besides, according to the elaboration likelihood model (ELM), under the condition of high involvement, the information receiver has strong motivation to carefully process information who would focus on persuasive information; under the condition of low involvement, the relevant motivation is weak, leading them more likely to pay attention to some non-core factors, such as the reliability of information sources (Petty and Briñol, 2011). In other words, more involved in social conflict events, the young people are better able to process core information and to express their views and understand different viewpoints in online public deliberation, so as to conduct rational discussions. Therefore, the study proposes hypothesis as follows:

H1: The event involvement degree positively affects the degree of online public deliberation.

#### 2.2.2 Inter-group emotional contagion and degree of online public deliberation

As a significant factor in the network discussion of social conflict events, emotion of an individual could be easily changed by group emotional contagion, which serves as an essential cause of group psychology in network discussion. For youth, the main group in cyberspace, they are also more likely to act due to emotional contagion. In the 1990s, researchers began to systematically elaborate and investigate the concept and theory of emotional contagion, during which intergroup emotion theory became an important theory to explain the formation and transmission of group emotions and interaction between individual emotions.

Additionally, the rise of the Internet and its technical characteristics make it easier for individuals to perceive large-scale group emotions, easily stimulating attitudes, emotional diffusion and resonance, and then letting them take or change actions (Smith et al., 2007). In fact, Stieglitz and Dang-Xuan (2013) have proved that emotional information is more likely to spread in the network, because emotional factors drive information re-sharing, which means emotion is actually one of the major motivations for individuals to participate in online public consultation. What's more, in this study, inter-group emotional contagion means that individuals' emotional experience is influenced or assimilated by the network emotions diffusion and contagion in social conflict events.

Moreover, social conflict events often elevate group emotions, among which the inter-group emotional contagion has a great impact on the group behavior. As a group behavior, public deliberation's degree will be affected by this kind of group emotion. On the one hand, at present, the expression on the network public platform mostly exhibits “emotional” and “irrational,” seeming difficult to reach the consensus expected by democratic deliberation. Besides, reason is considered to occupy the dominant position in public discussion, while although emotion can be the motivation to participate in public deliberation, it has a negative effect on the degree of public deliberation. However, with the deepening of the research, numerous scholars believe that public deliberation and its extended theory have evolved into over-rationalized concepts, which obscure or underestimate emotion in political interaction and citizens' various motivations of dealing with this political emotional interaction (Bickford, 2011). The emotional state itself is linked to the thinking and expression of the argument to make conversation and negotiation possible. That is, though inter-group emotional contagion is often used as a synonym for group polarization and irrationality, emotion is the driving force and maintenance agent of deliberation. Therefore, this study proposes the following research hypothesis:

H2: Inter-group emotional contagion positively affects the degree of online public deliberation.

#### 2.2.3 The mediating effect of selective exposure

As an important type of motivation, will is an internal psychological process that consciously controls and regulates behaviors to achieve predetermined goals. For instance, selective exposure stands as a typical manifestation of individual will in network information behavior. Individuals independently decide whether to expose and the degree of exposure by recognizing and evaluating information, involving active choice and control of their own behaviors which requires willpower to maintain and execute related behaviors. Specifically, audiences are more willing to contact what is consistent with or close to their existing positions and attitudes, while tending to avoid what is opposite or in conflict with. In result, in the face of social conflicts, selective exposure would affect the degree of online public deliberation.

In the online society, individuals transcend the limitations of time and space to communicate. Sunstein (2001) believes that netizens enjoy great autonomy, and it is easier for them to obtain the information they like and reject the information they do not like; at the same time, in online discussions, netizens are more likely to provide more arguments for their original tendencies. Thus, when netizens are selectively exposed to information that aligns with their interests and values, they are inclined to actively participate in discussions. Similarly, Towne and Herbsleb (2012) suggest that engaging participants in discussions relevant to their personal interests or abilities could enhance online public deliberation participation. All in all, although many scholars hold that selective exposure and the resulting collaborative filtering and information cocoon would make network negotiation prone to group polarization, rather than rational. However, there are relatively limited empirical studies on the effects of selective exposure to different information contents on individuals' political cognition and negotiation willingness (Liu and Liao, 2022). So based on social conflict events, this study proposes the following research hypothesis:

H3: Selective exposure positively affects the degree of online public deliberation.

Will is also the mediator of cognitive, emotional and behavioral coherence. From the individual, relevance and negative mood are both related to selective exposure behavior (Wang et al., 2024). Firstly, relevance refers to the degree of correlation between information and individual cognition and subjective feelings, which indicates that people often ignore irrelevant information and prefer relevant topics and information (Mummolo, 2016), further showing that the involvement degree serves as a significant reason for selective exposure. Secondly, negative mood are the possible consequences of inter-group emotional contagion, that is, individuals with negative mood would keep away from inconsistent information to coordinate cognition (Jean Tsang, 2019). Emotion would become people's standard of information screening.

Therefore, the involvement of social conflict events and the contagion of inter-group emotions will strengthen the selective exposure of individuals, enabling them to conduct public deliberation based on their own circumstances. This study proposes the following hypotheses:

H4: Selective exposure has a positive mediating effect between the event involvement degree and the degree of online public deliberation.

H5: Selective exposure has a positive mediating effect between inter-group emotional contagion and the degree of online public deliberation.

**Figure 1:**
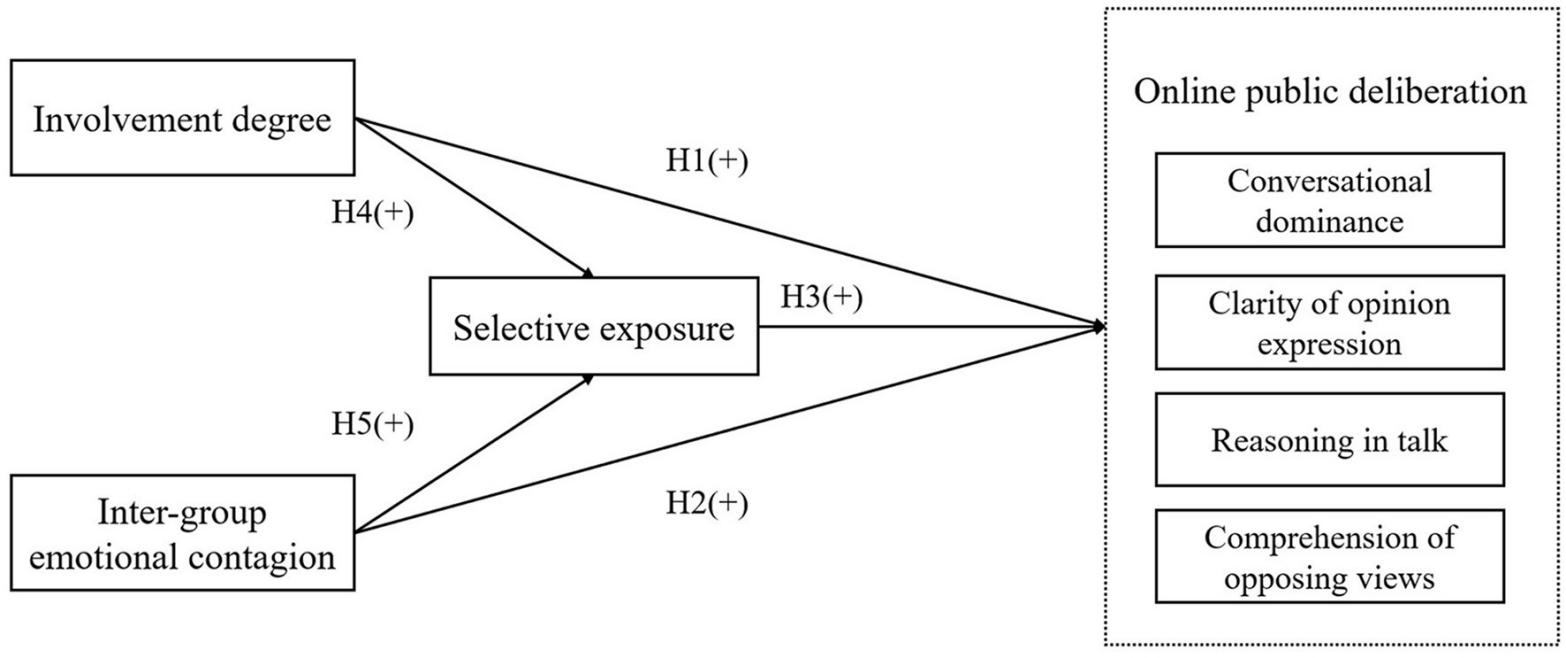
The research model of the degree of youth's online public deliberation.

From the perspective of psychological motivation, this study analyses the influencing mechanism of youth's online public deliberation degree in the context of social conflict events from three aspects of event involvement, inter-group emotional contagion, and selective exposure. The research model is shown in Figure 1.

### 2.3 Methods

#### 2.3.1 Participants and data collection

Before conducting the formal survey, we first determined the required sample size based on the margin of error and the confidence level. Using the Raosoft sample size calculator (http://www.raosoft.com/samplesize.html), we estimated that a minimum sample size of approximately 377 would be needed to achieve a 95% confidence level with a 5% margin of error. This figure served as the baseline minimum sample size for this study. Concentrating on how the event involvement degree and inter-group emotional contagion affect an individual's degree of online deliberation through selective exposure, this study distributed 160 pre-test questionnaires and 1,400 formal questionnaires on the platform of a Chinese professional survey company (i.e., Credamo). At the initial stage of the investigation, a formal questionnaire was formed after revising the content and form according to the results of the pre-test questionnaire. Besides, the age range of the participants refers to the definition of *young people* by the Communist Youth League of China (*Aged 14–28*), and combined with the screening conditions provided by the platform, the age range of the participants is finally determined to be “15–30 years old”. In detail, participants were asked to recall their experiences learning about social conflict events on social media and having public discussions with others. Finally, 1,092 participants completed the questionnaire with valid answers, with an effective rate of 78% according to quality control screening, in which the average age was 22.7 years old (Min = 15, Max = 30, *SD* = 2.92), the proportion of males was 40.5%, individual annual income mode ranged from 10,000 to 39,000. Moreover, SPSS and AMOS were used to analyse the data. According to the calculation results from Raosoft, with a sample size of 1,092, the margin of error is 2.88%, indicating that the sample is sufficiently large to support robust data analysis. The statistical tools employed in this study are SPSS and AMOS.

#### 2.3.2 Measures

In this study, nominal scales were used to collect demographic information of participants, which includes gender, education background and per capita annual income, with other major variables being measured by 7-Likert scale. Moreover, the measurement items were all based on the published scale developed or improved by scholars.

The independent variables of this study included the event involvement degree (EI) and inter-group emotional contagion (IEC). To begin with, the degree of event involvement refers to the study of Zaichkowsky and other scholars (1985), adopting three items for measurement, including “I think those social conflict events are closely related to me.” (1 = *strongly disagree* to 7 = *strongly agree, M* = 5.224, *SD* = 1.069, *Cronbach's alpha* = 0.745). Furthermore, inter-group emotional contagion was based on the measurement methods of Yang et al. (2016) and adopts three items, including “I will be affected by the collective emotions (i.e., anger, sympathy, hatred, guilt, etc.) of social conflict events on social media and jump into action.” (1 = *strongly disagree* to 7 = *strongly agree, M* = 3.516, *SD* = 1.403, *Cronbach's alpha* = 0.835).

Selective exposure (SE) stands as the mediating variable of this study, which is with reference to the measurement methods of Wang et al. (2018), adopting three items such as “I will choose to pay attention to the views on social conflicts events that are consistent with my values.” (1 = *strongly disagree* to 7 = *strongly agree, M* = 5.499, *SD* = 1.065, *Cronbach's alpha* = 0.754).

The degree of online public deliberation, the dependent variable of this study, measured online deliberative conversation behavior, according to the methods of Moy and Gastil (2006) and Wu et al. (2023), in which participants were asked to recall the last time they discussed social conflict events and related public issues with others on social media. What's more, the measurement is divided into four dimensions and eight items (1 = *strongly disagree* to 7 = *strongly agree*), and the four dimensions are as follows: (1) conversational dominance (CD), representative item: “I took the lead in the conversation.” (*M* = 2.957, *SD* = 1.289, *Cronbach's alpha* = 0.660) (2) clarity of opinion expression (COE), representative item: “I have clearly and directly stated my position.” (*M* = 4.798, *SD* = 1.274, *Cronbach's alpha* = 0.798) (3) reasoning in talk (RT), representative item: “I support my opinion by intelligent arguments.” (*M* = 5.472, *SD* = 1.033, *Cronbach's alpha* = 0.895) (4) comprehension of opposing views (COV), representative item: “I understand the reasons behind the other side's views.” (*M* = 5.113, *SD* = 1.125, *Cronbach's alpha* = 0.769), among which conversational dominance is the negative dimension of online public deliberation while the other three dimensions are positive.

## 3 Results

### 3.1 Correlation analysis, validity and reliability

In this study, the correlation analysis of the main variables was carried out first, and the results were shown in Table 1, according to which the correlation coefficients of all major variables were below 0.7, indicating that there was no obvious multicollinearity in the construction of variables (Tabachnick and Fidell, 2001). Moreover, the composite reliability (CR) of all variables ranged from 0.719 to 0.896, exceeding the standard of 0.6 (Fornell and Larcker, 1981); the factor loading of the measurement items ranged from 0.52 to 0.94; the average variance extracted (AVE) for each variable ranged from 0.502 to 0.812, which are greater than the recommended limit of 0.5 (Hair et al., 2016); the AVE square root values are all greater than the correlation coefficients among the variables. In summary, the reliability and convergent validity of each variable in this scale are satisfactory, and the discriminant validity of the scale meets the general requirements.

**Table 1:** Correlation coefficient and validity test.

**Variables**	**CR**	**AVE**	**1**	**2**	**3**	**4**	**5**	**6**	**7**
1.IEC	0.839	0.636	**0.798**						
2.EI	0.751	0.502	0.320^***^	**0.708**					
3.SE	0.759	0.512	0.376^***^	0.610^***^	**0.716**				
4.CD	0.719	0.580	0.208^***^	0.233^***^	0.241^***^	**0.762**			
5.COE	0.802	0.670	0.216^***^	0.362^***^	0.400^***^	0.484^***^	**0.818**		
6.RT	0.896	0.812	0.117^***^	0.287^***^	0.341^***^	0.308^***^	0.677^***^	**0.901**	
7.COV	0.779	0.640	0.129^***^	0.318^***^	0.354^***^	0.216^***^	0.375^***^	0.460^***^	**0.800**

^***^p < 0.001. Bold font is the square root of AVE. The rest is the Pearson correlation coefficient of each variable. EI, event involvement; IEC, inter-group emotional contagion; SE, Selective exposure; CD, conversational dominance; COE, clarity of opinion expression; RT, reasoning in talk; COV, comprehension of opposing views.

### 3.2 Descriptive statistics

Before examining the research questions, this study conducted a correlation analysis for all demographic variables and the degree of online public deliberation and the results are as follows:

In terms of specific dimensions, firstly, the age of young people was positively correlated with the clarity of opinion expression (*r* = 0.135, *p* < 0.001), reasoning in talk (*r* = 0.115, *p* < 0.001), and comprehension of opposing views (*r* = 0.082, *p* < 0.01). Secondly, the clarity of opinion expression of male youth group (*M* = 4.895, *SD* = 1.272) was higher than that of female youth group (*M* = 4.732, *SD* = 1.272); the reasoning in talk of male youth group (*M* = 5.615, *SD* = 1.012) was also higher than that of females (*M* = 5.374, *SD* = 1.036); the comprehension of opposing views of male youth group (*M* = 5.200, *SD* = 1.137) was also higher than that of females (*M* = 5.054, *SD* = 1.113). Thirdly, education background was positively correlated with conversational dominance (*r* = 0.132, *p* < 0.001) and comprehension of opposing views (*r* = 0.101, *p* < 0.001). Fourthly, the income of young people was positively correlated with the four dimensions of the degree of public deliberation, that is, the income is positively correlated with the conversational dominance (*r* = 0.085, *p* < 0.01), clarity of opinion expression (*r* = 0.192, *p* < 0.001), reasoning in talk (*r* = 0.207, *p* < 0.001), and comprehension of opposing views (*r* = 0.141, *p* < 0.001). Finally, compared with those living in provincial capital cities, municipalities, county-level cities, towns and rural areas, young people living in small and medium-sized cities had a higher degree of conversational dominance (*M* = 3.162, *SD* = 1.327, *p* < 0.001).

### 3.3 Model testing

The research model proposed in this study is as follows: the involvement degree in social conflict events and inter-group emotional contagion would jointly influence the degree of online public deliberation through selective exposure. Therefore, using the structural equation model (SEM) of AMOS 24 and adopting the maximum likelihood estimation, the overall fitting degree between the research model and the data was obtained. Besides, the fitting indexes, reference values and model fitting degree are shown in Table 2, and according to the permissible range (Zhang et al., 2020), the fitting degrees of χ^2^*/df* , RMSEA, GFI, NFI, TLI, CFI and AGFI and other indexes in this study all meet the requirements. More specifically, the RMSEA value of 0.041 indicates a low per-degree-of-freedom error rate, suggesting a good fit between the model and the observed data. A CFI above 0.90, along with a TLI exceeding 0.90, further indicates that the model performs better than the baseline model. In this study, the CFI value of 0.974 suggests that the theoretical model explains 97.4% of the covariance among variables. This indicates a high level of consistency between the theoretical construct and the observed data. Other fit indices, including GFI, AGFI, and NFI, also meet commonly accepted academic standards. Taken together, these indicators demonstrate a well-fitting model and provide robust empirical support for the hypothesized paths.

**Table 2:** SEM model-fit statistics.

**Index**	***χ^2^***	***df***	***χ^2^/df***	**GFI**	**AGFI**	**NFI**	**TLI**	**CFI**	**RMSEA**
Model indicator values	282.923	99	2.868	0.971	0.955	0.961	0.965	0.974	0.041
Cutoff criteria			1 < NC < 5	>0.9	>0.9	>0.9	>0.9	>0.9	< 0.08

**Figure 2:**
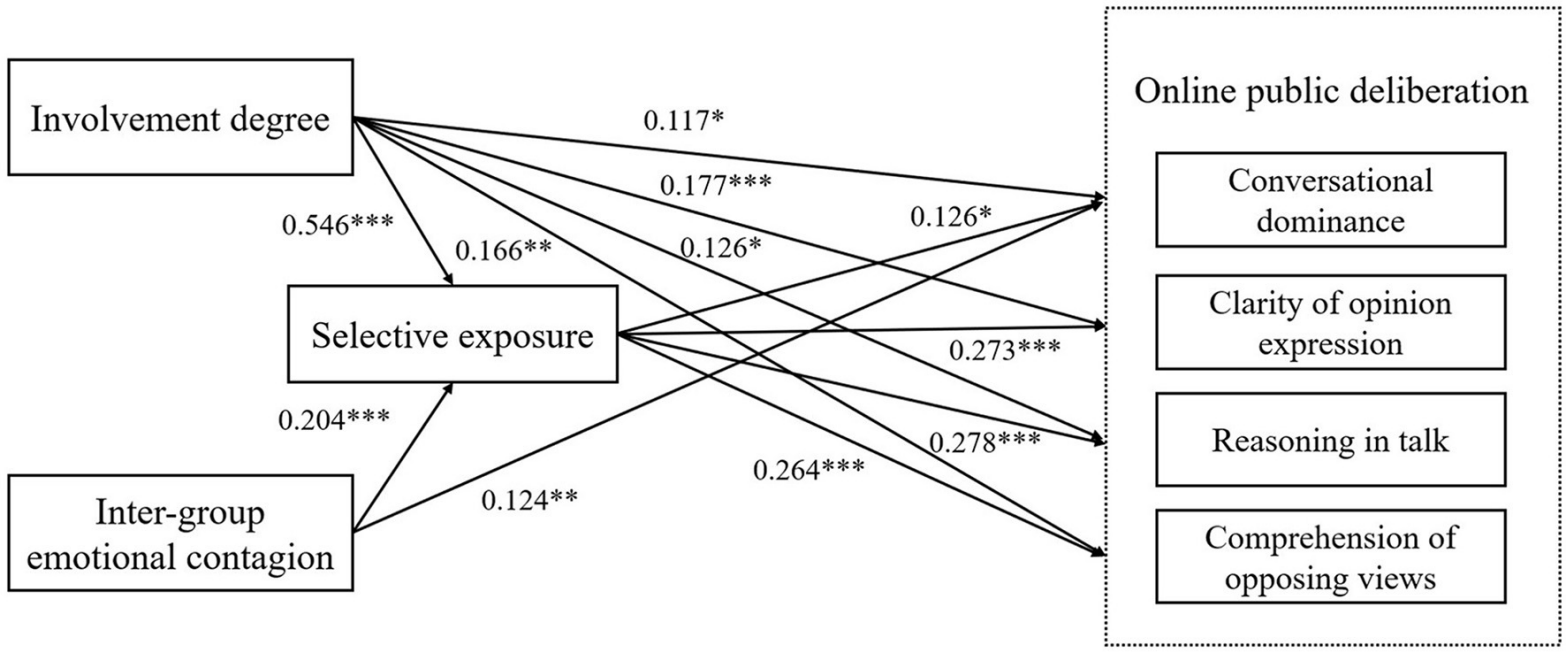
Relationship among various variables. The path coefficient is a standardized coefficient. ^***^p < 0.001, ^**^p < 0.01, ^*^p < 0.05.

Through AMOS 24, we examined the relationship between the event involvement degree, inter-group emotional contagion, selective exposure and online public deliberation (H1, H2 and H3). To start with, H1 holds that the event involvement degree positively affects the degree of online public deliberation and as shown in Figure 2, the path coefficient of the event involvement degree on the clarity of opinion expression was 0.177 (*p* < 0.001), reasoning in talk 0.126 (*p* < 0.05), and comprehension of opposing views 0.166 (*p* < 0.01), all of which have positive effects. Meanwhile, the event involvement degree positively affected conversational dominance, and the path coefficient was 0.117 (*p* < 0.05). Therefore, H1 is partially supported.

Then, H2 holds that inter-group emotional contagion positively affects the conversational dominance. As shown in Figure 2, the associations between inter-group emotional contagion and clarity of opinion expression, reasoning in talk and comprehension of opposing views did not reach statistical significance level (*p* > 0.05). And the path coefficient of inter-group emotional contagion on conversational dominance was 0.124 (*p* < 0.001), which had a positive effect, that is, H2 is not supported.

In addition, H3 posits that selective exposure positively affects the degree of online public deliberation. As shown in Figure 2, the path coefficient of selective exposure on the clarity of opinion expression was 0.273 (*p* < 0.001), the path coefficient on the reasoning in talk was 0.278 (*p* < 0.001), and the path coefficient on the comprehension of opposing views was 0.264 (*p* < 0.001), all of which have positive effects. At the same time, selective exposure also had a positive effect on conversational dominance, with a path coefficient of 0.126 (*p* < 0.05), that is, H3 is partially supported.

This study further applied Bootstrap to analyse the mediating effect of selective exposure (i.e., Bootstrap = 5,000, confidence interval = 95%), taking the degree of online public deliberation as the dependent variable, selective exposure as the mediating variable, the event involvement degree and inter-group emotional contagion as the independent variable, and investigated eight paths respectively, as shown in Table 3. The 95% confidence interval of each path does not contain 0, indicating that the mediating effect of selective exposure is significant, and H4 and H5 are supported.

**Table 3:** The mediating effect of selective exposure and significance test.

**Variable relationship**	**Estimate**	**S.E**.	**Bias corrected**		***p***	**Results**
			**Lower**	**Upper**		
EI → SE → CD	0.096	0.042	0.013	0.179	0.018	H4 supported
EI → SE → COE	0.194	0.044	0.114	0.288	0.000	
EI → SE → RT	0.191	0.043	0.113	0.284	0.000	
EI → SE → COV	0.125	0.036	0.060	0.203	0.000	
IEC → SE → CD	0.031	0.015	0.005	0.066	0.022	H5 supported
IEC → SE → COE	0.063	0.020	0.030	0.106	0.000	
IEC → SE → RT	0.062	0.020	0.029	0.107	0.000	
IEC → SE → COV	0.040	0.016	0.016	0.079	0.000	

Statistical significance is achieved when 0 is not included between the Lower and Upper. EI, event involvement; IEC, inter-group emotional contagion; SE, Selective exposure; CD, conversational dominance; COE, clarity of opinion expression; RT, reasoning in talk; COV, comprehension of opposing views.

## 4 Discussion

Based on national survey data and from the perspective of individual motivation, this study analyzed the differences in the degree of online public deliberation among young people in the context of social conflict events through the psychological process of cognition, emotion and volition. Besides, this study deepens the understanding of individual participation and psychological process in online public deliberation, broadens the occurrence scenarios and boundary conditions, providing new insight for the design of online public deliberation institutions and fields. On the whole, the positive dimensions of young people's degree of online public deliberation obtained higher scores, all above the average, among which the highest was the reasoning in talk (*M* = 5.472), while one of the negative dimensions, conversational dominance is lower than the average (*M* = 2.957). This suggests that Chinese youth perceive themselves as behaving relatively rationally in public discussions about social conflict events. Moreover, firstly, the study has found that the event involvement degree has a positive impact on the three dimensions of online public deliberation, but it also leads to a higher degree of conversational dominance. Secondly, inter-group emotional contagion only benefits in clarity of opinion expression, and has no impact on the improvement of public deliberation, resulting in higher conversational dominance. Finally, selective exposure, as an essential volitional expression of user regulation behavior, acts as a significant mediating role between individual cognition and emotion and online public deliberation.

### 4.1 Cognitive motivation dominates youth public deliberation

This study offers more support for the positive relationship between cognitive motivation and the degree of online public deliberation. Specifically, youth's involvement degree in social conflict events (*M* = 5.224) positively affects three positive dimensions of online public deliberation, which indicates that young people attach great importance to their connection to social events. It embodies their strong sense of social concern, the enthusiasm of and ability to participate in public affairs discussions. Furthermore, the youth stage stands as a key period for the formation of self-identity and values when young people usually start to think about their role, status and values in society, pay attention to social conflict events and have a strong willingness to express themselves, which is the manifestation of young people's shaping of their own identity. Then, young people also transform their self-cognition and self-efficacy into the ability to take part in public deliberation, hoping to be involved in the discussion of social conflict events with clear and wise views, patient listening and serious understanding.

In this study, the positive influence of the event involvement degree on the clarity of opinion expression and comprehension of opposing views is higher than that of reasoning in talk, which means in discussion young people tend to be more focused on expressing their positions and understanding opposing viewpoints while perceiving connection with social events. What's more, it also proves that in the case of high involvement, people may pay more attention to processing persuasive information, which would help them comprehensively understand issues, respect and tolerate contradictory views. It could facilitate deeper and productive discussions, rather than merely pursuing superficial logical rationality or consensus. However, on the other hand, it should be noted that if the event involvement degree continues to increase, young people would be more concentrated on their personal views and feelings in the discussion, ignoring the rational reasoning or logical analysis of the discussion. Of course, this circumstance is also connected to the defects of online public deliberation shaped in the social media environment. Namely, structural factors such as the anonymity of the network and the characteristics of text expression might restrict the improvement of the discussion rationality. For instance, some studies have pointed out that due to the anonymity of the Internet, the degree of politeness expressed by users may be reduced (Halpern and Gibbs, 2013). In addition, the expressive characteristics of short online texts and the use of memes would limit the constructive degree of discussion, so that even in the case of high involvement, young people may neglect to discuss with reasonable arguments and evidence, thus reducing the positive impact of the event involvement degree on the reasoning in talk.

Moreover, the study also found that the event involvement degree positively affects both online public deliberation and negative dimension (i.e., conversational dominance). In detail, the more involved in social conflict events young people are, the stronger willingness to express themselves they would have. In this situation, they usually tend to show their own stance through dominant expression. Besides, Chinese scholars Ma and Ma (2018), through the analysis of Internet public opinion on social conflict events, also proved that the result involvement is simultaneously related to the aggressive Internet expression and the consultative Internet expression, ignoring why opposing expression methods are integrated. In fact, from the point of view of democratic deliberation, it is not difficult to figure out how two opposing expressions can coexist, that is, in public deliberation, participants can make validity claims that challenge opposing parties. Additionally, Burkhalter et al. (2002) believe that reasonable public deliberation dialogue itself contains certain contradictions and conflicts, and participants should distinguish the contradictions around the theme and form a consensus in the discussion. In this study, the data also shows that the positive dimensions would also increase when the involvement degree increases, which reflects the better education level and civic literacy of contemporary youth. Although they have strong motivation to express their social concerns, they also realize that unilateral and strong expression does not act as the most effective way to solve the problem, so as to resist imposing their views on other participants. As a result, they are also willing to adopt negotiated expression and patient listening, solving problems through rational discussion and understanding, so dominant expression coexists with negotiated expression and comprehension of opposing views.

### 4.2 Inter-group emotion not conducive to youth public deliberation

In this study, inter-group emotional contagion (*M* = 3.516) has a significant positive impact on conversational dominance, indicating that under emotional motivation, young people attach importance to the expression of their own positions and viewpoints in public deliberation, which echoes Bickford (2011), Young (1996) and other scholars' views. They hold that emotion should not be excluded from reasonable political communication and public deliberation. Specifically, Bickford believes that the emotion constitutes habitual value judgments, and emotional beliefs play a central role in political communication. Similarly, while focusing on social events, young people feel the inter-group emotion, which can stimulate young people to express their ideas more actively and confidently to a certain extent, may promoting more effective communication. In addition, Hall (2007) believes that passion is inherent in public deliberation, and this dialectical relationship must be recognized if we want to deconstruct the duality between reason and emotion, so that emotions such as enthusiasm are an integral part of people's value judgments.

At the same time, Bickford (2011) also believes that “more emotional communication modes should not be simply privileged,” because with the encouragement of inter-group emotions, clarity of opinion expression of, reasoning in talk and counter viewpoint understanding have not been improved, and the interaction of young people on social media may be more inclined to emotional self-expression. A genuinely deliberative discussion is marked by the absence of conversational dominance, the presence of clear and reasoned argumentation, and mutual understanding among participants. Emotional contagion amplifies emotion-driven contributions, but not necessarily the quality of those contributions. In practice, when a group's mood is aroused, the most emotionally aroused individuals often seize the floor, and others feed off that energy which raises dominance but does not ensure arguments are clear or logically structured. Indeed, high emotion may overwhelm careful reasoning. In other words, contagion-driven discussions were more animated but not inherently more reasoned or transparent in argument.

In the context of social conflict events, conflicts themselves mean the existence of opposing opinions and unreachable consensus. Especially, young people are easily bound by group emotions and have a sense of empathy, turning originally rational discussion into some kind of emotional expression, while neglecting to link propositions and viewpoints with logical arguments and express them clearly in the discussion. Besides, the lack of efforts to understand and take into account the arguments and points of view expressed by the participants in the deliberation is detrimental to the conduct of public deliberation. Formal and deliberate negotiation conversations need to be complemented by careful and sympathetic listening (Burkhalter et al., 2002), because only when a negotiation dialogue is thoughtful could participants have a clearer understanding of the reasons and values behind the surficial opposing views. Therefore, at a time of heightened social tension caused by social conflicts, it is necessary to guide young people to maintain respect and listen to each other, so as to have reasonable discussions and conversations and improve the degree of public deliberation.

### 4.3 The mediating effect of selective exposure

Selective exposure (*M* = 5.499), as the volitional motivation of regulatory behavior, positively affects the three positive dimensions of online public deliberation of young people, which indicates that young people would increase their willingness to take part in public deliberation and be able to conduct reasonable discussions and deliberations with a clearer perspective after they are exposed to events that are close to their existing positions, views and attitudes. As a result, young people could purposefully choose social events of interest or concern, which may raise their motivation to obtain relevant information. What's more, the generally connected network society has expanded people's exposure to different viewpoints, or “at least not limited people's exposure” (Stromer-Galley, 2003), so do the young people. They could have diverse observation perspectives on social events and a deeper understanding of the subject of negotiation, resulting in a more insightful idea and a better ability to understand opposing opinions.

However, selective exposure also increases conversational dominance, that is, speakers tend to be more confident and feel that they are in charge of the conversation when it comes to events that correspond with their own perceptions, emotions, and evaluations. This also indicates that individuals tend to seek out echo chambers in response to emotionally charged, conflictual, and psychologically engaging events. Internet technology driven by algorithm and data has strengthened the echo chamber effect, and the probability of people being exposed to information similar to the existing position has greatly increased, which would consolidate the inherent cognition, and even produce group polarization, leading to more extreme views than the original. On social media, the information individuals encounter largely mirrors their own behaviors and interests, as algorithms simply personalize content based on prior engagement. Besides, this will also make the speeches of the participants tend to be domineering, which does harm to the development of public deliberation. To sum up, as a volitional motivation, selective exposure is a relatively stable psychological process in the information behavior of young people which would have an impact on the degree of online public deliberation.

Furthermore, this study found that both the event involvement degree and inter-group emotional contagion had a positive influence on selective exposure, through the mediating role of selective exposure, it positively affected all dimensions of online public deliberation. Individual cognition and emotion serve as crucial causes of selective exposure. In detail, when young people are affected by collective emotions or feel connected with social conflicts, they are more likely to spend time, and are more willing to participate in discussions that are consistent with their attitudes, in which they can clarify their views, understand contradictions, and rationally debate. In other words, the level of online public deliberation is not only determined by cognition or emotion alone, but is also closely relevant to the individual's volitional ability, especially selective exposure.

However, this intermediary influence mechanism would also raise the conversational dominance of young people in online public deliberation, which is not conducive to the improvement of the degree of deliberation. Driven by cognition, emotion and will, young people tend to form a discourse leading and emotional resonance mode of conversation.

### 4.4 Group differences and future prospects of youth online public deliberation

This study also proved the difference between the degree of online public deliberation of different youth groups, providing a certain reference basis for improving the public deliberation ability of young people in the social media environment. Specifically, with the increase of age, the degree of online public deliberation would also continue to improve, and there was no significant relationship between conversational dominance and age. In the process of growing up, young people's personal experience, knowledge level and civic quality continue to upgrade, so they will participate in public deliberation more mature and stable.

In addition, the higher the income of young people, the higher the three positive dimensions of online public deliberation degree, but also lead to higher conversational dominance. In fact, youth from higher-income or more educated families tend to have better connectivity, digital literacy, and argumentation skills, enabling more active and assertive participation, but low-income youth groups might be limited by realistic conditions, facing digital barriers and lacking time and opportunity to participate in deliberation. Educators need to pay more attention to youth from lower socioeconomic backgrounds.

Moreover, the higher the education level, the higher the comprehension of opposing views, but also the higher the conversational dominance; the clarity of opinion expression, reasoning in talk and comprehension of opposing views of male youth group are higher than those of female youth group, but there is no significant difference in the conversational dominance. But previous studies suggest that conversational dominance is closely linked to gender and professional knowledge. For example, Leet-Pellegrini (1980) found that men and experts are more dominant than women and non-experts in negotiation and discussion and there is also more interference by investigating the offline public deliberation of college students. In fact, this study confirmed the influence of educational background on conversational dominance of young people in the network environment, but there was no significant difference between different genders. At the same time, young male group also show a more rational way of dialogue and empathy, which may be associated with the way of expression of men, who tend to express themselves in longer language (Hogg, 1985). Jaidka et al. (2019) illustrated that in the network environment, if the length of online blog posts is doubled, the occurrence of impolite expressions could be reduced, increasing the level of civility and making public discussion more productive. Meanwhile, previous studies suggest that online harassment and gender bias can further discourage female youth from speaking up or elaborating arguments fully (Fox and Tang, 2017). In summary, different gender ways of thinking and expression may lead to differences in the degree of deliberation, but it does not mean that the role of female youth groups in public deliberation is missing which deserves greater attention.

## 5 Limitation and future prospects

In the end, there is still some room for improvement in our research. In terms of sample composition, there is a gap between the gender structure of respondents and the 52nd Statistical Report on Internet Development in China, so that more attention should be paid to the difference between universality and particularity in the promotion of research conclusions. Meanwhile, this study did not include items that directly measure conflictual or insulting language. As a result, we cannot make sure whether participants' arguments, even if well-structured, may have included hostile or aggressive remarks. This limits our ability to assess the true tone and constructiveness of the discussions. Future research should consider adding measures that capture the presence of conflict or incivility in participants' communication.

Future studies could further segment the nature and sources of motivation, and explore their impact on the degree of public deliberation. For example, this study treated selective exposure only as a mediator but did not explore its potential role as a moderator. Conversations are generally more constructive when individuals engage within echo chambers composed of like-minded others. However, when participants are exposed to environments dominated by conflicting or alternative viewpoints, discussions often break down and become less deliberative. At the same time, an alternative situation may arise: exposure to like-minded sources would intensify polarization and reduce reflexivity, thereby reinforcing cognitive bias and attitudinal extremity. Conversely, encountering cross-cutting views can foster bias correction and broaden perspective-taking, thereby enriching deliberative quality. These phenomena require empirical validation in future research.

Given that the cross-sectional nature of the study only elucidates correlations among variables, future research could employ longitudinal data or experimental methods to confirm the causal relationships it suggests. Besides, self-reported measures can capture whether youth participate and with what kind of motivations, but they cannot fully reveal how deliberation unfolds in real time. Including qualitative insights into youth discourse could provide a more holistic view of online deliberation.

More specifically, compared to other countries, and given China's unique cultural context, a next step could be to conduct a cultural analysis of how Chinese youth engage in public discussion. Confucian and collectivist norms emphasizing harmony cultivate a conversational style in which dissent is expressed supportively and indirectly, in stark contrast to the confrontational and analytical approach typical of Western individualistic contexts.

In addition, the concept of public deliberation, which originated in the era of traditional mass communication, has also changed with the evolution of media and space. It is necessary to continue reflecting on the emerging value connotations, new subject participation, and new evaluation dimensions of public deliberation in the networked society.

## 6 Conclusion and value

All in all, from the perspective of individual intrinsic motivation, this study systematically examines the direct and mediating effects of involvement, group emotion, and self-will on the degree of online public deliberation, further detailing how individual actors engage in and influence public deliberation in social media. The research findings suggest that involvement positively influences the level of online public deliberation. However, similar to intergroup emotional contagion, it may also lead to an increased degree of conversational dominance. Furthermore, selective exposure functions as a significant mediator between individual cognition, emotion, and online public deliberation.

The social media platform in the network society is a two-way accelerator. For one thing, it enhances the potential of rational conversation between people and provides new opportunities for democratic participation and deliberation. For another, it also magnifies the prejudice, indifference, anger and even hatred among people, spreading a lot of false information and even triggering social unrest. Accordingly, correctly guiding young people to engage in political participation in cyberspace and stimulating the endogenous power of democratic deliberation is a key part of building a community of shared future in cyberspace. Moreover, public deliberation is an inclusive problem-solving process that provides citizens with the opportunity to make meaningful judgments on public issues. Although a single conversation could rarely resolve social differences, with the increasing number of participation and the passage of time, public deliberation can promote the development of social democracy by improving the public's ability to judge the complexity of things, ultimately affecting the development and decision-making process of events.

## Data Availability

The raw data supporting the conclusions of this article will be made available by the authors, without undue reservation.
